# Development of Cortical Networks under Continuous Stimulation

**DOI:** 10.3389/fnmol.2017.00018

**Published:** 2017-01-31

**Authors:** Ophir Orenstein, Hanna Keren

**Affiliations:** ^1^Network Biology Research Laboratory, Electrical Engineering, Technion - Israel Institute of TechnologyHaifa, Israel; ^2^Department of Physiology, Biophysics and Systems Biology, Technion - Israel Institute of TechnologyHaifa, Israel

**Keywords:** neural networks, development, stimulation, synchronization, cultures

## 1. Introduction

Neural networks develop naturally under continuous reciprocal interaction with the environment, which results in early life experiences having an immense impact on adult capacities to function and adapt to the environment (Vygotsky, 1978; Black et al., 1987; Greenough et al., 1987; Sur et al., 1988; Pallas et al., 1990; Jones and Greenough, 1996; Markham and Greenough, 2004; Marom, 2015). It is well established, for example, that sensory inputs during development play an essential role in shaping brain circuitry and neuronal connectivity, as was shown by the classical studies of Hubel and Wiesel in which sensory deprivation enhanced fundamental alterations of primary sensory areas (Hubel and Wiesel, 1977; Hubel et al., 1977; Greenough et al., 1987; Sur et al., 1988). However, the underlying neural principles and mechanisms which impact such developmental interactions remain largely unknown. This state-of-the-art environment-development problem, calls for controlled, multi-level experimental access to neural networks development over a wide range of temporal and spatial scales. The major aim of this work, therefore, is to provide an experimental database for studying how external inputs encountered during development can affect the mature aspects of neuronal network activity—implemented in neural networks developing *in-vitro*.

Cultures of cortical networks comprise a relevant experimental model for characterizing development of neural activity, without being affected by predefined constraints and intervening processes (Katz, 1993; Wong, 1993; Marom and Shahaf, 2002). The developmental stages and their corresponding time frames are surprisingly similar to those observed *in vivo*. When a population of cortical neurons is extracted from a rat newborn and allowed to develop for a couple of weeks outside the brain, it forms a network that exhibits complex spontaneous activity. During this process, uncorrelated firing appears at the end of the first week in culture, then evolving to synchronous activations observed as early as 9–12 days *in-vitro* (Gross, 1979; Habets et al., 1987; Stenger and McKenna, 1994; Kamioka et al., 1996; Corner et al., 2002; Marom and Shahaf, 2002; Morin et al., 2005; Eytan and Marom, 2006; Wagenaar et al., 2006). Synchronous events frequency increase to an average rate of ca. 0.1 Hz, in dependence on a rapid growth in networks connectivity, both in terms of number of connections and connection length, between 5 and 10 days *in-vitro* (Maeda et al., 1995; Marom and Shahaf, 2002; van Pelt et al., 2005; Wagenaar et al., 2006; Napoli et al., 2014).

By applying electric stimulation to these networks, it is possible to evoke a synchronized response that propagates across extended parts of the network (Maeda et al., 1995; Pinato et al., 1999; Jimbo et al., 2000; Marom and Shahaf, 2002; Wagenaar et al., 2004). Such stimulation has been shown to simulate modulatory effects of sensory inputs, as inducing neural plasticity and changes in network connectivity (Jimbo et al., 1998, 1999; Maeda et al., 1998; Tateno and Jimbo, 1999; Rolston et al., 2007; Bologna et al., 2010; Keren and Marom, 2014). This model provides a well-controlled setting for studying impacts of external inputs during development, as addressed by previous studies in which activity was sampled along development *in-vitro* following sparse stimulation phases (Wagenaar et al., 2006; Bologna et al., 2010).

**Figure 1:**
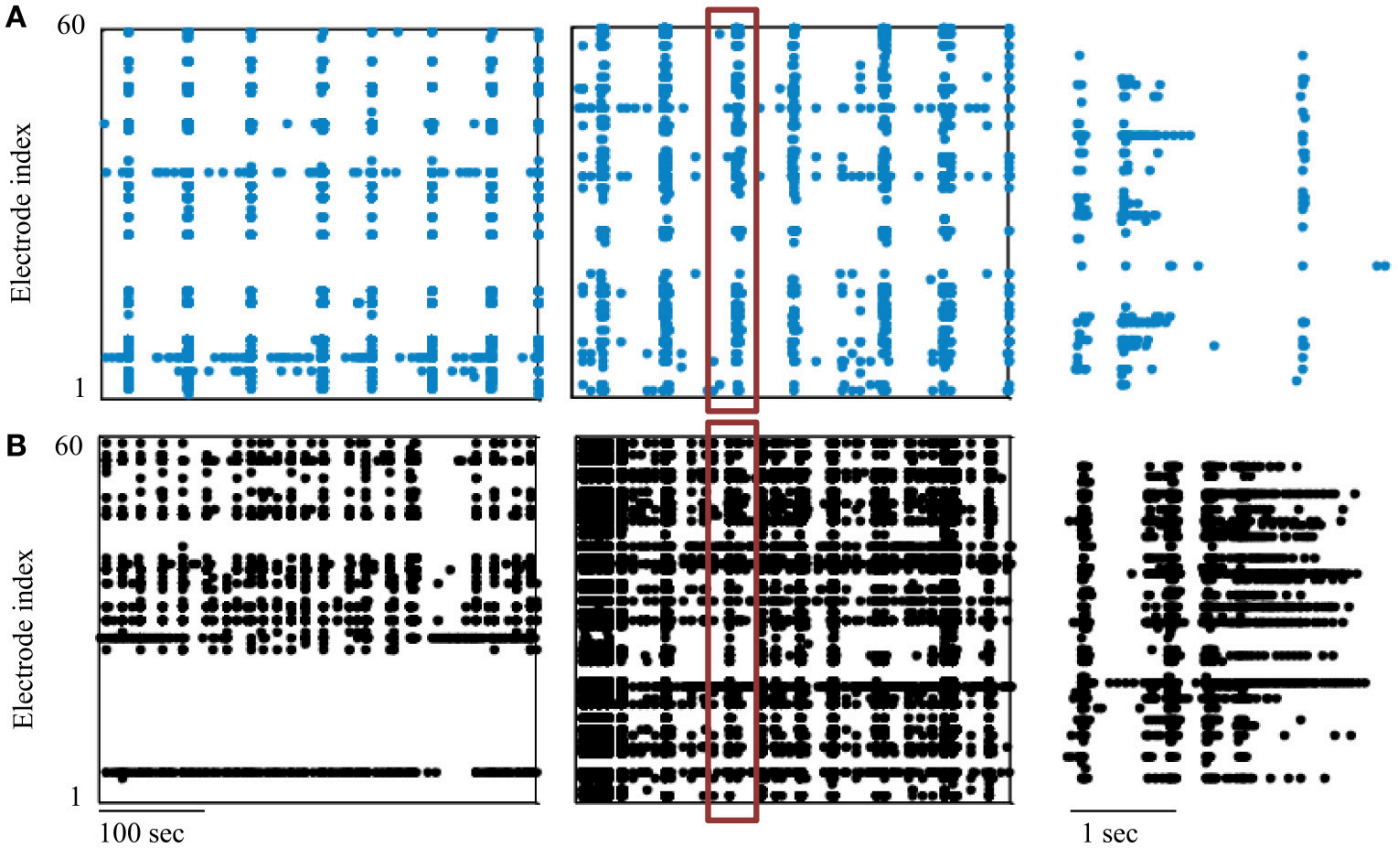
**Neural networks development data**. Segments of activity recorded during the ninth day *in-vitro* (DIV) are presented in **(A)** for two non-stimulated networks and in **(B)** for two networks exposed to continuous stimulation. Each point depicts a single spike detected in one of the electrodes (indexed in the vertical axis); the right-hand panel shows the boxed synchronous event at higher temporal resolution.

This work, extends these studies in the direction of continuous recording and stimulation of cortical networks in the course of development, as of the second day *in-vitro*. The experimental database is comprised of six networks developing while exposed to continuous stimulation, as well as an additional two non-stimulated networks, similarly monitored. Stimulation was applied alternately from two spatially distinct sources with a uniform distribution of inter-stimulus-intervals ranging from 6 to 14 s. Ultra-slow perfusion of culture medium was used to ensure this ongoing long-range experimental setting. Examples given in Figure 1, present extracts of recorded activities during the ninth day *in-vitro* of two non-stimulated networks (A) and two continuously stimulated ones (B). Note that in four of the six stimulated networks, parts of the sampled region could not be detected to a certain extent, possibly due to deficiencies in technical monitoring, thus are absent from synchronized activity (synchronization properties, however, are still apparent and measurable in these cases, as exemplified in Figure 1B, left).

**Figure 2:**
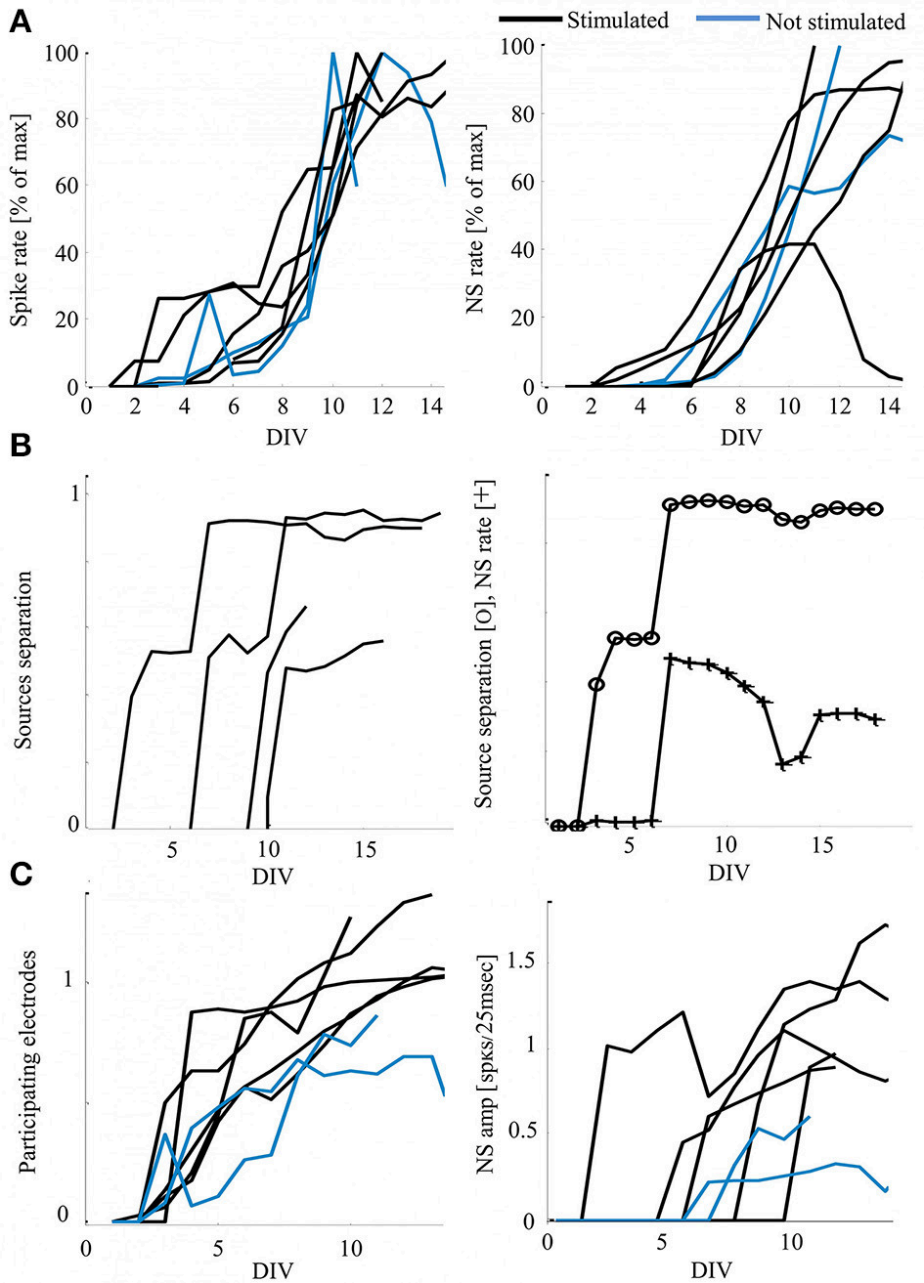
**Development of activity—functionality measures and possible impacts of stimulation. (A)** Development of activity rate measures, calculated as the mean value per day *in-vitro* (DIV) normalized to the individual maximal value for presentation clarity (Hz). Each curve represents a single network, color-coded according to the developmental conditions. **(B)** Examples of stimulated networks demonstrating increasing distance between recruitment orders evoked by the two stimulation sources. Order vectors are comprised of the first spikes of the first five electrodes; the distance is calculated as the average of a pair-wise distance matrix using Levenshtein Edit Distance metric (Shahaf et al., 2008). Right: an example of the possible correspondence between the classification of stimulation sources (distance) and the response rate, scaled for presentation clarity (*CC* = 0.83). **(C)** Stimulated networks (black) show increased number of participating electrodes per day *in-vitro* (left, mean values of 0.51 and 0.7 for non-stimulated and exposed to stimulation networks, respectively, with *p* < 0.005 by *t*-test) and synchronous events amplitude (right, mean values of 0.26 and 0.62 for non-stimulated and exposed to stimulation networks, respectively, with *p* < 0.005 by *t*-test). Normalization across networks is to the number of electrodes active in each network during the last day of recording (active is considered as > 5 spikes).

We chose to address this data using synchronization as a system variable, relevant for neural network function and activity and driven by external inputs. The development of synchronization in particular, has been suggested as the underling process for the fine-tuning of mature, functional neural circuits (Yuste et al., 1992; Katz and Shatz, 1996; Zhang and Poo, 2001; Leinekugel et al., 2002; Corlew et al., 2004; Khazipov et al., 2004; Kilb et al., 2011). Our experiments outline the gradual emergence of network synchronization between days 6 and 9, as shown in Figure 2A. Moreover, in this context, functionality can be approached through the capacity to classify (i.e., discriminate between) different inputs, a primitive that stands at the basis of cognitive capabilities. The development of efficient classification between two stimulation sources, estimated by recruitment orders of spike representations, is described in Figure 2B (left), as is its possible dependence on synchronization rate (example given in Figure 2B, right). Exposure to stimulation during development, is suggested to impact networks' mature activity, as demonstrated by a potential increase in synchronous activity properties of relative amplitude and duration (evident in Figure 1B), as well as by an increase of active electrodes participation during synchronization (see Figure 2C, reflecting the count of spikes during synchronization by each electrode). It should be noted that each network was cultured in a separate batch, repeating the same procedure using different animals.

Fruitful directions for research might include questions as diverse as the dependence of mature propagation orders of neurons across the network on initial activity-connectivity profiles (e.g., we found that units generating early activities are recruited later across the network in mature synchrony propagation, reflecting early activation convergence to a “follower” type of activation) and aspects of activity rhythms occurring along a scale of hours to days. Experimental extensions toward full reciprocal interaction with the environment by closing the loop on a key measure that is relevant for function and that is affected by external inputs, could modify the experienced environment and enhance underlying alterations of activity occurring during development (Shahaf and Marom, 2001; Wagenaar and Potter, 2004; Wagenaar et al., 2005; Arsiero et al., 2007; Rolston et al., 2010; Keren and Marom, 2014). Overall, we believe that the provided database and proposed interpretations have the potential to contribute insights regarding environmental interaction with the course of neural development.

## 2. Materials and methods

### 2.1. Cell preparation

Cortical neurons were obtained from newborn rats (Sprague-Dawley) within 24 h after birth using mechanical and enzymatic procedures described in earlier studies (Marom and Shahaf, 2002). Rats were anesthetized by CO_2_ inhalation according to protocols approved by the Technion's ethics committee. The neurons were plated directly onto a substrate-integrated multi electrode array and allowed to develop into functionally and structurally mature networks over a period of 2–3 weeks, while being continuously recorded and stimulated. The number of plated neurons was of the order of 450,000, covering an area of about 380 mm^2^. The preparations were perfused continuously (ultra slow perfusion with an exchange rate of about 0.06 ml/h) with MEM supplemented with heat-inactivated horse serum (5%), glutamine (0.5 mM), glucose (20 mM), and gentamycin (10 μg/ml), and maintained in an atmosphere of 37°C, 5% CO_2_ and 95% air.

### 2.2. Electrophysiology

An array of Ti/Au extracellular electrodes, 30 μm in diameter, spaced 500 μm from each other and located in the center, was used (MultiChannelSystems, Reutlingen, Germany). A commercial amplifier (MEA-1060-inv-BC, MCS, Reutlingen, Germany) with frequency limits of 150–3000 Hz and a gain of x1024 was obtaining data. Data was digitized using data acquisition board (PD2-MF-64-3M/12H, UEI, Walpole, MA, USA). Each channel was sampled at a frequency of 16 kHz. The insulation layer (silicon nitride) was pre-treated with polyethyleneimine (Sigma, 0.01% in 0.1 M Borate buffer solution). Data acquired was analyzed using Matlab (Mathworks, Natick, MA, USA).

### 2.3. Experimental procedure

Following cell plating, networks were kept in an incubator with controlled conditions as described above for 24–48 h, and then placed on the recording setup for the entire duration of the experiment (9 DIV being the shortest experiment and 43 DIV the longest). Six networks were stimulated via two alternating, spatially distant electrodes (29 and 49, in this order), with inter-stimulus-intervals ranging between 6 and 14 s and uniformly distributed (0.12 Hz ± 0.04). Each recording day included an hour with no stimulation, when spontaneous activity data was obtained. Two experiments depict spontaneous activity only, recorded along the development (experiments #1 and #8). In all cases, recording was stopped once every 3 days for up to 30 min for maintenance of the perfusion system.

### 2.4. Analyses

Action potentials were detected by threshold crossing which is defined separately for each of the recording channels at the beginning of an experiment (6 × standard deviation of a 2 s voltage trace). A refractory period of 6 ms was considered. Electrical activity detected often originates from several sources, typically 3-2 neurons, as each recording electrode is surrounded by several cell bodies. Detection of synchronous events was performed on-line by threshold crossing of summed action potentials within 25 ms. Exact threshold was determined relative to 25% of the active electrodes (typically the value of 20 action potentials).

### 2.5. Overview and format of the database

The database can be accessed through the following link: https://figshare.com/s/5cff3bb4e25b814c1459. It contains separate datasets (“projects”) for each of the experiments (files within are numbered accordingly, from 1 to 8). Each dataset comprises text files numbered by their recording order. The text files can be opened using various interfaces, such as text editors, Excel, Matlab or Mathematica and include a table of time stamps (in seconds) of all spikes (in the first column); the corresponding electrode indexes for each of the spikes (row-wise in the second column); and the shape of each spike (as of the third column; Each column represents a sample unit equals to 0.0625 msec and voltage units are of 0.42 μV). Loading these files to Matlab might provide two variables: *textdata*—with the two leftmost columns representing the spike time stamps and electrode index, respectively; and *data*, which describes the spike shape corresponding to each of the spikes indicated by the file textdata (note that the first two rows of textdata are excluded).

Beyond the 60 electrodes of the recording array, index 61—represents time stamps of stimulation, and index 64 represents the detection of a synchronous event (the time of threshold crossing in an on-line detection). Electrodes 63 and 62 depict internal blanking signals and should be omitted (typically, the first two spikes—rows, of each data file).

The time stamps across consecutive files are continuous up to the reset of recording for maintenance, which occurred about every 3 days (which caused a restart of time stamps to zero). A continuous activity times vector can be generated by adding the last time stamp prior to the reset to all the consecutive times of the following data files. The stimulation electrode index (29 and 49, alternating) is given in a separate file named StimulusIndex. This file comprises 25,920 stimuli and was reused at each reset of recording. An hour of no stimulation was integrated daily for the stimulated networks, indicated by an index of “0” in the StimulusIndex file (as of stimulation 25,559). The respective stimulation times are given in the file StimulusTimes. For simplification of analyses, an additional file named Days provides the organization of all experiments files by the recording day *in-vitro* (an Excel file that presents the range of file numbers belonging to a day, with days indicated by the row number and each experiment organized in a separate sheet). The spatial layout of all electrodes is provided in a file named Array, in which electrode indexes are schemed by their spatial location.

Through-out the recordings, artifact synchronous activities may occur, characterized by an immediate precise synchrony across the entire culture, a typical event in such experimental systems (possibly due to a drop of vaporized liquid). These events can simply be excluded from analyses by detecting the occurrence of a synchronous event in all electrodes in a time window as short as a couple of milliseconds.

## Author contributions

OO performed the experiments, initial analyses, and created the database, HK performed the analyses and wrote the manuscript.

### Conflict of interest statement

The authors declare that the research was conducted in the absence of any commercial or financial relationships that could be construed as a potential conflict of interest.
